# Prognostic performance of a series of model for end-stage liver disease and respective Δ scores in patients with hepatitis B acute-on-chronic liver failure

**DOI:** 10.3892/mmr.2014.1983

**Published:** 2014-02-25

**Authors:** YUN-HAO XUN, JUN-PING SHI, CHUN-QING LI, DAN LI, WEI-ZHEN SHI, QING-CHUN PAN, JIAN-CHUN GUO, GUO-QING ZANG

**Affiliations:** 1 Department of Liver Diseases, Hangzhou Sixth People’s Hospital/Xixi Hospital of Hangzhou, Zhejiang University of Traditional Chinese Medicine, Hangzhou, Zhejiang 310014, P.R. China; 2 Department of Infectious Diseases, Shanghai Sixth People’s Hospital, Shanghai Jiaotong University, Shanghai 200233, P.R. China

**Keywords:** acute-on-chronic liver failure, hepatitis B, models for end-stage liver disease, Δ score, prognosis

## Abstract

The present study aimed to compare the short-term prognostic performance of a series of model for end-stage liver disease (MELD) and respective delta (Δ) scores scoring systems in a population with acute-on-chronic hepatitis B liver failure (ACHBLF), and to investigate the potential effects from antivirals. A total of 77 patients with ACHBLF of mean age 46 years, 82% male, with 58.4% receiving antivirals, were recruited for this study. The Δ scores for MELDs were defined as the changes one week after admission. Thirty-eight (49%) patients (22 treated with antivirals) died within three months. The mean MELD and ΔMELD scores of the survival group were 19.5±4.4 and 0.2±3.7 respectively, and those of the mortality group were 23.5±5.5 and 7.9±6, respectively. The area under the receiver operating characteristic curve (AUC) for MELD, integrated MELD (iMELD), MELD with the addition of serum sodium (MELD-Na), updated MELD (upMELD), MELD excluding the international normalized ratio (INR; MELD-XI), United Kingdom MELD (UKMELD) and their Δ scores were 0.72, 0.81, 0.77, 0.69, 0.65, 0.77 and 0.86, 0.83, 0.83, 0.82, 0.79 and 0.79, respectively. iMELD and MELD-Na significantly improved the accuracy of MELD (P<0.05). A cut-off value of 41.5 for the iMELD score can prognose 71% of mortalities with a specificity of 85%. In each pair of models, the Δ score was superior to its counterpart, particularly when applied to patients with MELD ≤30. Decreased accuracy was observed for all models in the subset of patients treated with antivirals, although their baseline characteristics were comparable to those of untreated patients, while iMELD, MELD-Na and respective Δ models remained superior with regard to the predictability. The iMELD and MELD-Na models predicted three-month mortality more accurately, while the Δ models were superior to their counterparts when MELD ≤30; however, their performance was altered by antivirals, and thus requires optimization.

## Introduction

As a common fatal liver disease, acute-on-chronic liver failure (ACLF) was not well-defined until the concept was revised by the Asian Pacific Association for the Study of the Liver (APASL) in 2008 (1). However, a number of important issues, including prognostic assessment, still require clarification. Considering the high short-term mortality (~50–90%) observed in absence of liver transplantation (LT), it is undoubtedly important to improve the accuracy of prognosis for patients with ACLF. Prognostic models, developed for donor liver allocation and validated based on patients with end-stage liver disease (ESLD), may not be applicable to patients with acute-on-chronic hepatitis B liver failure (ACHBLF) (2). In fact, liver-specific scoring systems such as the model for end-stage liver disease (MELD), were recommended by APASL for ACLF patients only as weak evidence with level 3b and grade C (1). There is currently no evidence that MELD-based models perform equally well in ACLF. The MELD system, considered a milestone for prognosis of ESLD, has numerous advantages over other, less extensively evaluated scoring systems in terms of objectivity and performance stability, although some refinement is required to improve its suboptimal accuracy (3); for example, addition of serum sodium, as well as other variables, improve the predictive accuracy of MELD in some settings (4). To date, only a few studies on heterogeneous populations used different diagnostic criteria for ACLF and ACHBLF to validate the potential of MELD, MELD with the addition of serum sodium concentration (MELD-Na) or weekly measurement of MELD combined with initial MELD score (5–10). More validation studies on prospective cohorts using the latest diagnostic criteria are urgently required. Given the inherent pathogenesis for ACLF, an acute event, superimposing on the underlying chronic liver disease, is the real determinant of the outcome (1). Its prognosis is more difficult than that of acute or chronic liver failure (11). Therefore, a dynamic, and not a single initial assessment, as the one provided by the delta (Δ) score, is expected to provide more valuable information on the prognosis of ACLF, as recently evidenced in preliminary results from retrospective cohort studies on ACHBLF (8,10) and alcoholic ACLF patients (12). However, the real merits of this type of dynamic assessment need to be thoroughly studied and the time interval prior to repeating each score evaluation remains to be identified. Antiviral treatment with nucleos(t)ide analogs (NUCs) has been proposed as a basic therapeutic approach for patients with ACHBLF (1), but whether this treatment interferes with the prognostic accuracy is unknown. It was reported however that the short-term mortality, the predictive target of prognostic models, may be reduced by antivirals (13).

In mainland China, ~80–90% of ACLF cases have been attributed to hepatitis B virus (HBV) infection, which causes ~22, 600 deaths annually and remains an important challenge (14,15). In this context, the present study aimed to identify the most suitable scoring system by comparing, using the latest diagnostic criteria, the short-term prognostic performance of a MELD scoring series [MELD and its derivatives: updated MELD (upMELD), integrated MELD (iMELD), end-stage liver disease excluding the international normalized ratio (INR; MELD-XI), MELD-Na and United Kingdom MELD (UKMELD)] (4) and their respective Δ scores in a prospective cohort of ACHBLF patients. The potential effects of antiviral treatment on the prognostic accuracy of these models were also investigated.

## Materials and methods

### Patients

Adult patients with ACHBLF were recruited prospectively from April 1, 2009 to March 31, 2010 in the Hangzhou Sixth People’s Hospital (Hangzhou, China), a tertiary centre where LT is unavailable. Patients were excluded from the study if diagnosed with hepatocellular carcinoma, coinfection with HIV/HCV, bile duct obstruction, if they were orally receiving anticoagulants or presenting coexisting system disorders such as chronic kidney disease. Patients under an artificial liver support system intervention or receiving fresh frozen plasma were also excluded. This study conformed to the Helsinki Declaration of 1975 and was approved by the Ethics Committee of Hangzhou Sixth People’s Hospital. Written informed consent for inclusion in the trial was obtained from all patients.

### Diagnostic criteria

Chronic HBV infection was diagnosed as persistent infection with hepatitis B virus for >6 months. Detection of HBV markers in all patients was performed using ELISA kits (Abbott Laboratories, Abbott Park, IL, USA) at admission stage. The criteria used for the diagnosis of ACLF were based on the guidelines described by APASL (1). Briefly, these were acute hepatic insults manifesting as jaundice (serum bilirubin ≥5 mg/dl), coagulopathy [international normalized ratio (INR) ≥1.5] and the occurence of complications such as ascites and/or encephalopathy within 4 weeks in a patient previously diagnosed or undiagnosed chronic liver disease.

### Data collection and follow-up

Data concerning the demography, clinical, and laboratory variables were prospectively recorded at admission. The parameters used for the scoring of prognostic models were assessed every week during the hospitalization and every month after the hospital discharge, if the patient survived. All individuals were followed for at least 3 months after hospital discharge or until death. Antiviral treatment with NUCs was performed after receiving the informed consent of the subjects regarding the potential benefits and risks of the use of antivirals. The method for grouping patients by antiviral treatment was thus based on the participants’ intentions and not on randomization.

### Management of patients

Conventional support treatment was applied to all individuals. The main procedures included intensive care monitoring, lactulose and high-calory supplement treatment, and bowel wash. Albumin supplement, antibiotics, and proton pump inhibitors were used when necessary.

### Calculation of scores

The MELD, upMELD, iMELD, MELD-XI, MELD-Na and UKMELD scores were evaluated at admission and calculated by the following formulas (mg/dl for creatinine and bilirubin and mEq/l for serum sodium), respectively: MELD score = 11.2 × ln (INR) + 9.57 × ln (creatinine) + 3.78 × ln (bilirubin) + 6.43 (16); upMELD score = 1.266 ×ln (1 + creatinine) + 0.939 × ln (1 + bilirubin) + 1.658 × (1 + INR) (17); iMELD score = original MELD score + (age ×0.3) − (0.7 × Na) + 100 (18); MELD-XI score = 5.11 × ln (bilirubin) + 11.76 × ln (creatinine) + 9.44 (19). MELD-Na score = MELD score -Na − [0.025 × MELD × (140 − Na)] +140 (20); UKMELD score = [(5.935 × ln (INR) + (1.485 × ln (creatinine)) + (3.13 × ln (bilirubin)) − (81.565 ×ln (Na))] + 435 (21). Δ scores for these models were defined as the magnitude of change 1 week after admission, or were based on the last valid data for patients who died within the 1st week.

### Data analysis and statistics

Continuous variables were expressed as the mean ± standard deviation. Comparisons between groups were performed by Student’s t-tests, and by χ^2^ tests for categorical parameters. The Cox proportional hazards model was used to estimate the hazard ratio of predictors for the 3-month mortality, and comprised parameters such as age, gender, antiviral treatment, and all laboratory test results and MELD scores. Parameter antiviral treatment was excluded when grouped with antivirals. The area under the receiver operating characteristic curve (AUC) was used to compare the prognostic accuracy of models applied on all subjects or subsets of these, stratified by antiviral treatment or by the type of initial MELD model applied. An AUC >0.7 was considered to be clinically relevant (8,22). The Delong test was used to compare the AUCs of MELD derivatives with the traditional MELD, ΔMELDs with their counterparts, and ΔMELD derivatives with ΔMELD (23). Optimal cut-off values were derived from the Youden’s index J = (sensitivity + specificity − 1) (22). A P<0.05 (from two-sided tests) was considered to indicate a statistically significant difference. Statistical analysis was performed using SPSS software version 16.0 software (SPSS, Inc., Chicago, IL, USA).

## Results

### Patients’ characteristics

A total of 98 patients with ACHBLF were recruited. Of these, 21 were excluded (19 under artificial liver support system intervention, 1 transferred for LT, and 1 dropped out), and the remaining 77 individuals were included in the analyses. A total of 45 (58.4%) patients received NUCs as antivirals [18 lamivudine, 15 entecavir, 8 adefovir, and 4 patients with lamivudine resistance (rtM204I) received monotherapy and adefovir].

As shown in Table I, 38 (49%) patients deceased within 3 months after admission, with a median survival time of 17.5 (range, 5–83) days, and 5.3% (2/38) of deaths occurred within the first week. The mean age of all subjects was 46 (18~65) years, males were more commonly affected than females with a ratio of 4.5:1. Twenty-five (32.5%) patients had preexisting cirrhosis. The mean viral load was 6.0±2.3 (log_10_ copies/ml) and 31 patients (40.3%) were positive for HBeAg. Fifty-six (72.7%) patients presented complications during hospitalization, the most common of which was spontaneous bacterial peritonitis (41/77, 53.2%).

Compared to patients who survived (survival group), the ones who deceased (death group) were older, and had longer INR and higher bilirubin levels (P<0.05 for all). Severe hyponatremia (<126 mEq/l), as a known mortality predictor for ESLD (24), was found only in one case (1.4%), although the serum sodium level was lower in the group of deceased compared to that of patients who survived (P<0.05). Neither alanine aminotransferase (ALT), nor viral parameters or treatment with antivirals were significantly different between the two groups. In addition, a higher incidence of complications (P<0.05) and an increased trend for preexisting cirrhosis (39.5 vs. 25.6%, P=0.195) were observed in the death group. As expected, the death group showed significantly higher scores in all MELD scoring systems compared to the survival group, and the differences between the two groups were more marked in Δ scores than in their counterparts (P<0.05 for all, Table I). The median score was 21 (range, 11~33) for MELD and 3 (range, −6~21) for ΔMELD. At the time of the second evaluation, the proportions of initial MELD scores that increased, remained stable and decreased were 69% (53), 3% (2) and 29% (22), respectively.

Regarding the comparison of patients based on the antiviral treatment, all demographic and clinical characteristics as well as the MELD and ΔMELD scores were comparable (P>0.05 for all), except for the ALT level (451 vs. 882 IU/l, P=0.013). The percentage of patients who deceased within three months and the median survival time for the patients receiving or not antivirals were 48.9 vs. 50%, and 22.5 vs. 11.5 days, respectively (Table II).

### Prognostic factors associated with 3-month mortality in the Cox proportional hazards model

Three factors, namely age, bilirubin level and INR, were identified to independently increase the 3-month mortality risk in all subjects and in those with MELD score ≤30. INR was the only risk factor for the subset of patients receiving antivirals, while age combined with the creatinine level and INR were identified as risk factors for the subset of patients who were not treated with antivirals (Table III).

### Different performance of prognostic models for the 3-month mortality assessment

**Figure 1:**
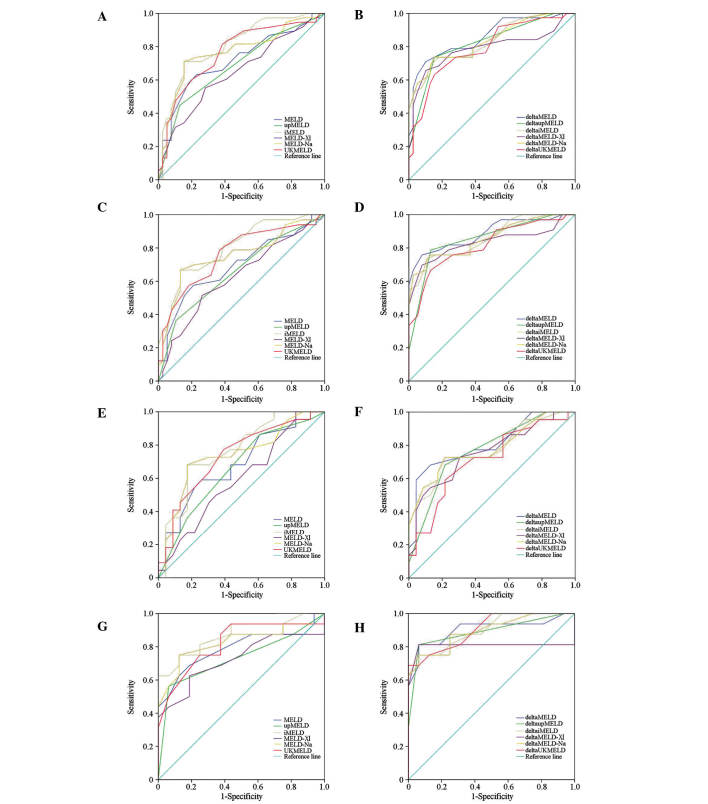
Comparison of area under receiver operating characteristic (ROC) curves (AUC) for model for end-stage liver disease (MELD) and respective delta scores for the 3-month mortality assessment in acute-on-chronic hepatitis B liver failure (ACHBLF) patients with different characteristics. (A,B) all, (C,D) MELD score ≤30, (E,F) treated with antivirals, and (G,H) not treated with antivirals. For an explanation of MELD models, see previous tables.

The AUC was estimated to be >0.5 for all progostic models (0.647–0.807, P<0.05 for all) applied on all subjects; this value corresponds to a consistently appropriate sensitivity and specificity. The iMELD score had the highest AUC of 0.807 (95% CI, 0.71–0.905) with a sensitivity of 71.7% and a specificity of 84.6% for an optimal cut-off value of 41.5. It was followed by MELD-Na, UKMELD, MELD, upMELD and MELD-XI in terms of performance. Similar results were observed when model scores were compared at the same cut-off value and patients with MELD score >30 were excluded, with only MELD-XI failing to predict the 3-month mortality in this subset (AUC=0.628, P=0.065). In comparison to the AUC of MELD (0.717 for all subjects and 0.695 for those with MELD score ≤30), prognostic accuracy was increased in the iMELD and MELD-Na (P<0.05 for all), decreased in the MELD-XI, and remained equivalent in the UKMELD and upMELD models (Table IV and Fig. 1A and C).

In each pair of models, the AUC of the Δ score was higher compared to that of its counterpart, with the respective values >0.7 for all six models applied on all patients (0.789–0.859) and on those with MELD score ≤30 (0.818–0.888). Furthermore, ΔMELD-XI performed better compared to the respective, poorly performing MELD-XI model, in the subset of patients with MELD ≤30, and similarly, the prognostic accuracy of the ΔupMELD score was significantly improved compared to the upMELD score in the same subset (P<0.05 for both). Except for the slightly reduced accuracy of ΔMELD-XI compared to ΔMELD in all patients (P=0.044), the performance of the other four Δ models was very high and statistically equal to that of ΔMELD in both subsets, all patients and patients with MELD ≤30 (Table IV and Fig. 1B and D).

### Effects of antivirals on the prognostic performance of MELDs

When the subjects were stratified by antiviral treatment, a consistent decrease in accuracy was observed for each model in the group treated with antivirals. With regards to the corresponding AUCs in the group not treated with antivirals (0.725–0.871), both MELD-XI and upMELD failed to predict the 3-month mortality (P>0.05), and fewer MELD models had an AUC >0.7 in the group treated with antivirals (0.579–0.762). In line with their performance for all subjects, MELD-Na and iMELD had relatively higher AUC values, but no statistical difference was detected for the comparison to MELD (AUC=0.762, 0.736 and 0.66, respectively, P>0.05 for both comparisons) (Table IV and Fig. 1E and G).

**Figure 2:**
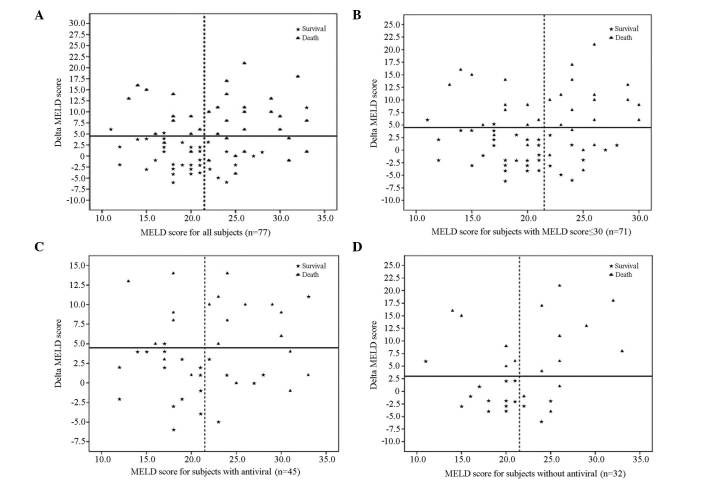
Comparison of MELD and delta (Δ)MELD scores between survival and death groups with different characteristics.

As for the Δ models, an improvement in prognostic accuracy was observed for each model in patients treated with antivirals, with the highest AUC value (0.806) coming from ΔMELD and similar values from the other models (0.711–0.782). In patients not treated with antivirals, the Δ scores for the MELD series of models consistently resulted in high AUC values, as high as 0.904 (Table IV, Fig. 1H). Although no significant differences were observed in the AUCs between ΔMELDs and their counterparts in both subsets of patients, among all of those with MELD ≤30 (P>0.05 for each, Table IV), a higher number of patients with a poor clinical outcome were accurately classified based on optimal cut-off values. This favorable ability of ΔMELDs for classification was just reflected by the comparison of ΔMELD and MELD scores between the survival and the death group with different characteristics (Fig. 2).

## Discussion

Based on the latest criteria for diagnosis of ACLF described by the APASL (1), this study validated the prognostic ability of MELD, derivative models and their respective Δ scores in a population of ACHBLF patients with different characteristics, the value of which in the prognostic performance of the tested models was assessed [except for gender, omitted due to the tedious calculation it requires (25)]. From the direct comparison of performance of these different models within the same cohort, several important findings were obtained.

First, comparing the performance of MELD scores in predicting the 3-month mortality indicated that among the six MELD-based models, MELD-Na and iMELD and especially the latter, perform better than the traditional MELD. Since it is equally convenient to calculate the score of each model by using formulas available on websites or a given worksheet, it is necessary to identify the most accurate score to meet the aforementioned requirements in outcome prognosis. Based on the AUC values, MELD showed moderate accuracy in our study, similarly to previous reports (5,6,8,10). Thus, this score is clinically relevant but its suboptimal sensitivity and specificity need to be further improved, the related shortcomings also shown in previous studies of populations with similar clinical features (5,6,8,10). The different cut-off values used for MELD scoring system in other studies (6,8,10) are possibly due to the use of different diagnostic criteria and time-points chosen for scoring. The level of serum bilirubin (≥5 mg/dl) required for the definition of ACLF (1) is lower than the one measured in these studies (≥10 or 17.6 mg/dl) and a strictly initial assessment at admission but not the possible delayed detection in a retrospective study (5) would result in a lower MELD score and consequently, a lower cut-off value. Based on the optimal cut-off value derived from the standard method (22), a MELD score at admission as low as 21.5 is sufficiently high to alert on the need of closely monitoring these patients, which results in a higher number of validations required for MELDs in ACHBLF cohorts when the unified system for the definition of the disease is used.

Similarly to the need for MELD optimization in the prognosis of ESLD (3,4), adjustments are also needed to test how applicable this model is in Chinese populations with ACHBLF (10). In the present study, we observed an advantage for iMELD, in addition to the established and confirmed herein merit of the MELD-Na model. Incorporating natrium in combination with age, the main risk factor for mortility in this cohort, yielded the highest AUC in the MELD series of models, which indicates that this approach might be more promising compared to those adopted in current practice. Additional advantages of iMELD and MELD-Na are expected in populations with higher proportions of hyponatremic patients. A disadvantage of less accuracy for the MELD-XI model was observed in the following comparison. This poor performance can be partially explained by the predominant impact on mortality of the INR risk factor. INR was shown to be an independent predictor in the Cox proportional hazards model analysis for all subjects and any subsets of these. It is a well-known determinant of hepatic synthesis and one of the mandatory markers for defining liver failure (1); thus INR should not be neglected in MELD score assessment analyses.

Second, the advantage of using Δ scores over their respective MELDs was demonstrated in the ACHBLF population, with more prominent merits in patients with MELD ≤30 and those treated with antivirals. As variations in the results of repeated measurements of the MELD score were observed (26), ΔMELD has been evaluated in several populations, including ACHBLF populations with retrospective design and populations of ACLF caused by alcohol (8,10,12,27). Given the prompt need for dynamic evaluation of ACLF compared with the relatively more stable ESLD and acute liver failure, a Δ score for each of the MELD derivatives was introduced in this study based on previous definitions of ΔMELD (26,27) and ΔMELD-Na (10). As expected, the Δ score was superior to its counterpart in each pair of models, as for instance shown by the marked difference in these scores between the death and the survivor group, where AUC, sensitivity and specificity values associated with the ΔMELD scores were higher compared to those correspondingly original MELDs. A clinically relevant AUC >0.7 was observed for each Δ score in all subjects, and was further improved in those with MELD score ≤30, with an improvement observed even for the generally poorly-performing model MELD-XI. Moreover, the differences among the MELD models were attenuated by the delta approach, providing statistically comparable AUCs. Therefore, it is necessary to score the prognostic models repeatedly, facilitated by the fact that daily intensive care monitoring is indispensable in ACLF, and the score calculation can be easily repeated (1,4).

In addition to the merits of ΔMELD model shown in other studies (8,10), the time interval prior to the repetition of scoring was explored in this study. In our opinion, a shorter time of 1, but not 2, weeks is suitable for populations where early deaths occur [5.3% (2/38) of deaths occurred within the first week and 22.7% (5/22) within the second week in another study (8)]. A time-period of 2 weeks is indeed required for predicting the percentage of patients surviving following medical treatment (1,8). If the intention of the study is to predict the poor clinical outcome, a time-period as short as 1 week is suitable for detecting the changes in MELD and the derived scores. Nevertheless, a few patients with poor outcome may be clearly predicted and it was not possible to calculate a MELD score for them due to subsequent death, thus a shorter interval combined with initial scoring may represent a rational option for ACHBLF. Still, the optimal cut-off values for various ΔMELD models to predict short-term mortality remain to be determined.

Third, an interesting result was obtained from the comparison of performance of prognostic models between patients treated or not with antivirals, which revealed that all models have a consistently decreased accuracy for the group of patients treated with antivirals, although their baseline characteristics were comparable. As one of the most important therapeutic interventions, antiviral treatment with NUCs is recommended in consensus by hepatologists, so as to repress the replication of HBV in ACHBLF patients (1,11,28). In practice, this approach is adopted to a limited extent in China because of the associated high cost, insurance, required informed consent procedures, etc. It is thus necessary to clarify the potential effects of this type of therapy on the prognostic assessment. Although stratification was based on the patients’ wish to be treated with NUCs, comparable results were obtained, except for the serum ALT levels. All demographic, clinical, and laboratory variables were comparable between the two groups, which indicates that reliable comparisons are feasible. It was surprising that antivirals failed to improve the short-term outcome in the studied ACHBLF population. Further studies are required to explain this result, since the variable NUC sources and the small size of the studied population may have limited the power to address this issue in the current context. In addition, controversial results on this issue have been reported in other studies (13,29,30).

Compared to the concordant and clinically relevant AUCs associated with the group not treated with antivirals, both the original and the related ΔMELD scores were lower for the group treated with antivirals. The prognostic accuracy of MELD declined to <0.7, MELD-XI and upMELD failed to predict the 3-month mortality, and the remaining three models had weak AUCs similar to those observed in the analysis of all patients. Notably, the respective ΔMELD scores were improved, therefore, repeated evaluation of MELDs appears to be more crucial than antiviral treatment and sufficient to improve the prognostic performance of a model in this setting. The results from the Cox proportional hazards model analysis suggested that the differences in the effects of antiviral treatment may result from the differences among patients for certain mortality risk factors. The individual outcome for certain patients may be affected by various NUCs, thus affecting the predictive abilities of factors such as age and creatinine level in patients treated with antivirals. As a proof of concept, the underlying beneficial or deleterious effects of different NUCs on cytotoxic T-cell activity and mortality risk determinants of equal importance to HBV replication make it impossible to improve the clinical outcome of patients treated with antivirals in some conditions (1,8,29).

A number of limitations to this study need to be mentioned. The small sample size, the provenance of patients from a single health centre, and importantly, the small number of individuals with hyponatremia, limited the power to evaluate the prognostic performance of the tested models. The potential effects of various NUCs need to be addressed in the future, since their efficacy in reducing mortality from ACHBLF is potentially not comparable (29). Finally, even the best-performing prognostic model has limited predictive ability in practice. Thus, the information provided by a model should only be used as a supplement to other available information during the decision-making process for a given individual (31).

In summary, with regards to the predictive ability of MELD and associated Δ scores for the 3-month mortality of ACHBLF patients, iMELD and MELD-Na perform better than the traditional MELD, and a cut-off value of 41.5 for iMELD can identify 71% of deaths with a specificity of 85%. In each pair of models, the Δ score assessed within a 1-week interval is superior to its counterpart, and the advantage is more notable in the subset of patients with MELD ≤30, as well as in those treated with antivirals. However, the performance of all models is altered by antiviral treatment, thus highlighting the need for optimization and more detailed analyses in the future.

## Figures and Tables

**Table I:** Baseline characteristics of all subjects.

Variables	All patients (n=77)	Survival group (n=39)	Death group (n=38)	t/χ^2^ test	P-value
Age (years)	46±11	42±11	50±9	−3.35	0.001
Male, n (%)	63 (81.8)	33 (84.6)	30 (78.9)	0.416	0.519
Survival time (days)	-	-	17.5 (5–83)	-	-
Antiviral treatment	45 (58.4)	23 (59.0)	22 (57.9)	0.009	0.923
Cirrhosis	25 (32.5)	10 (25.6)	15 (39.5)	1.680	0.195
Complications	56 (72.7)	18 (46.2)	38 (100)	28.135	0.000
SBP	41 (53.2)	14 (35.9)	27 (71.1)	9.555	0.002
Other infections	19 (24.7)	5 (12.8)	14 (36.8)	5.975	0.015
HE	25 (32.5)	3 (7.7)	22 (57.9)	22.123	0.000
HRS	11 (14.3)	1 (2.6)	10 (26.3)	8.867	0.003
GI bleeding	4 (5.2)	1 (2.6)	3 (7.9)	1.110	0.292
HBeAg positivity	31 (40.3)	18 (46.2)	13 (34.2)	1.141	0.285
HBV DNA (log_10_ copies/ml)	6.0±2.3	5.9±2.2	6.1±2.4	−0.463	0.645
ALT (IU/l)	630.3±691.9	639.9±747.4	620.4±639.8	0.123	0.903
Albumin (g/l)	33.7±4.3	34.5±4.9	32.9±3.6	1.579	0.119
Sodium (mEq/l)	136.8±4.8	138.1±3.1	135.4±5.8	2.516	0.015
<126 n (%)	1 (1.4)	0 (0)	1 (2.9)	1.133	0.287
Bilirubin (μmol/l)	250.6±118.3	217.5±105	284.6±122.9	−2.578	0.012
Creatinine (μmol/l)	74.2±26.2	69.5±15.2	79.1±33.6	−1.603	0.115
INR	2.0±0.7	1.8±0.5	2.2±0.8	−2.692	0.009
Platelets (×10^3^/mm^3^)	104.5±60.2	107.6±64.1	101.3±52.8	0.457	0.649
Score					
MELD	21.4±5.3	19.5±4.4	23.5±5.5	−3.556	0.001
ΔMELD	4.9±63	0.2±3.7	7.9±6.0	−6.819	0.000
upMELD	5.0±0.8	4.8±0.7	5.3±0.8	−3.023	0.003
ΔupMELD	0.5±0.8	0.0±0.5	0.9±0.7	−6.459	0.000
iMELD	39.4±7.9	35.4±6.8	43.5±6.7	−5.300	0.000
ΔiMELD	6.1±8.2	1.6±5.0	10.7±8.3	−5.800	0.000
MELD-XI	19.9±4.7	18.7±4.1	21.2±5.0	−2.399	0.019
ΔMELD-XI	3.6±5.1	1.0±2.9	6.2±5.6	−5.086	0.000
MELD-Na	22.9±5.7	20.3±4.7	25.5±5.5	−4.411	0.000
ΔMELD-Na	4.4±5.9	1.0±3.7	7.8±5.8	−6.018	0.000
UKMELD	45.1±4.5..	43.2±3.5..	47.0±4.6..	−4.908	0.000
ΔUKMELD	3.5±4.8	1.2±3.3	5.9±4.9	−4.929	0.000

SBP, spontaneous bacterial peritonitis; HE, hepatic encephalopathy; HRS, hepatorenal sydrome; GI, gastrointestinal; ALT, alanine aminotransferase; INR, international normalized ratio; MELD, model for end-stage liver disease; upMELD, updated model for end-stage liver disease; iMELD, integrated model for end-stage liver disease; MELD-XI, model for end-stage liver disease excluding INR; MELD-Na, model for end-stage liver disease with the addition of serum sodium; UKMELD, United Kingdom MELD.

**Table II:** Comparison of baseline characteristics among patients treated or not with antivirals.

Variables	Patients treated with antivirals (n=45)	Patients not treated with antivirals (n=32)	t/χ^2^ test	P-value
Mortality, n (%)	22 (48.9)	16 (50.0)	0.009	0.923
Survival time (days)	22.5 (5–70)	11.5 (7–83)	−0.814	0.416
Age (years)	46±10	44±12	0.794	0.430
Male, n (%)	37 (82.2)	26 (81.2)	0.012	0.913
Cirrhosis	10 (25.6)	15 (39.5)	0.037	0.847
Complications	34 (75.6)	22 (68.8)	0.437	0.509
SBP	27 (60.0)	14 (43.8)	1.984	0.159
Other infections	10 (22.2)	9 (28.1)	0.351	0.554
HE	15 (33.3)	10 (31.2)	0.037	0.847
HRS	7 (15.6)	4 (12.5)	0.143	0.706
GI bleeding	2 (4.4)	4 (6.2)	0.124	0.725
HBeAg positivity	18 (40.0)	13 (40.6)	0.003	0.956
HBV DNA (log_10_ copies/ml)	6.3±2.3	5.6±2.1	1.357	0.179
ALT (IU/l)	451.1±495.1	882.2±844.7	−2.588	0.013
Albumin (g/l)	33.0±7.8	34.8±3.4	−1.841	0.070
Sodium (mEq/l)	136.2±5.4	137.6±3.8	−1.314	0.193
<126 n (%)	1 (2.4)	0 (0)	0.767	0.381
Bilirubin (μmol/l)	236.0±119	271±115	−1.292	0.200
Creatinine (μmol/l)	76.0±23.2	71.6±30.2	0.726	0.470
INR	2.1±0.8	2.0±0.5	0.399	0.691
Platelets (×10^3^/mm^3^)	98.4±54.1	113.1±67.8	−1.056	0.294
Score				
MELD	21.4±5.6	21.5±5.0	−0.124	0.902
ΔMELD	4.1±5.2	3.8±7.6	0.189	0.850
upMELD	4.9±0.9	5.2±0.7	−1.218	0.227
ΔupMELD	0.4±0.7	0.6±0.8	−0.932	0.355
iMELD	40.0±8.0	38.5±7.8	0.834	0.407
ΔiMELD	6.1±7.5	6.1±9.2	0.021	0.984
MELD-XI	19.9±4.8	20.0±4.6	−0.122	0.903
ΔMELD-XI	3.2±4.8	4.1±5.6	−0.779	0.439
MELD-Na	23.1±6.0	22.6±5.4	0.331	0.741
ΔMELD-Na	4.4±5.2	4.3±6.9	0.039	0.969
UKMELD	45.3±5.1	44.8±3.5	0.405	0.687
ΔUKMELD	3.6±4.8	3.3±4.7	0.272	0.786

SBP, spontaneous bacterial peritonitis; HE, hepatic encephalopathy; HRS, hepatorenal sydrome; GI, gastrointestinal; ALT, alanine aminotransferase; INR, international normalized ratio; MELD, model for end-stage liver disease; upMELD, updated model for end-stage liver disease; iMELD, integrated model for end-stage liver disease; MELD-XI, model for end-stage liver disease excluding INR; MELD-Na, model for end-stage liver disease with the addition of serum sodium; UKMELD, United Kingdom MELD.

**Table III:** Prognostic factors associated with the 3-month mortality in the Cox proportional hazards model.

Subjects	n	Variables	Hazard ratio (95% CI)	Wald test	P-value
All	77	Age	1.045 (1.012–1.078)	7.464	0.006
		Bilirubin	1.004 (1.001–1.007)	5.523	0.019
		INR	1.423 (0.953–2.124)	2.973	0.085
MELD score ≤30	71	Age	1.060 (1.024–1.098)	10.953..	0.001
		Bilirubin	1.005 (1.002–1.008)	9.348	0.002
		INR	2.769 (1.398–5.484)	8.538	0.003
With antivirals	45	INR	1.835 (1.162–2.898)	6.773	0.009
Without antivirals	32	INR	2.182 (1.036–4.596)	4.214	0.040
		Age	1.076 (1.015–1.141)	6.075	0.014
		Creatinine	1.026 (1.007–1.045)	7.526	0.006

MELD, model for end-stage liver disease; INR, international normalized ratio; CI, confidence interval.

**Table IV:** Predictive abilities of different prognostic models for acute-on-chronic hepatitis B liver failure (ACHBLF) patients.

Prognostic models	AUC (95% CI)	P-value	Cut-off value	Sensitivity (%)	Specificity(%)	P-value^a^	P-value^b^	P-value^c^
MELD	0.717 (0.600–0.833)^d,h^	0.001	21.5	63.2	76.9	0.062	-	-
ΔMELD	0.859 (0.776–0.943)^d,i^	0.000	4.5	71.1	89.7	-	-	-
	0.695 (0.569–0.820)^e,h^	0.005	21.5	57.6	78.9	0.019	-	-
	0.888 (0.808–0.968)^e,i^	0.000	4.5	75.8	82.1	-	-	-
	0.681 (0.523–0.838)^f,h^	0.038	21.5	59.1	73.9	0.244	-	-
	0.806 (0.676–0.936)^f,i^	0.000	4.5	68.2	87.0	-	-	-
	0.791 (0.625–0.957)^g,h^	0.005	21.5	68.8	71.2	0.337	-	-
	0.902(0.785–1.019)^g,i^	0.000	3.0	81.2	93.8	-	-	-
upMELD	0.687(0.567–0.806)^d,h^	0.005	5.5	44.7	87.2	0.070	0.312	-
ΔupMELD	0.823 (0.728–0.917)^d,i^	0.000	0.5	73.7	84.6	-	-	0.165
	0.661 (0.533–0.789)^e,h^	0.020	5.5	36.4	89.5	0.013	0.323	-
	0.854 (0.763–0.945)^e,i^	0.000	0.5	78.8	86.8	-	-	0.190
	0.660 (0.500–0.820)^f,h^	0.066	-	-	-	0.302	0.632	-
	0.770 (0.632–0.908)^f,i^	0.002	0.5	68.2	78.3	-	-	0.338
	0.732 (0.550–0.915)^g,h^	0.025	5.5	56.2	93.8	0.145	0.119	
	0.891 (0.769–1.012)^g,i^	0.000	0.5	81.2	93.8	-	-	0.784
iMELD	0.807 (0.710–0.905)^d,h^	0.000	41.5	71.1	84.6	0.799	0.020	-
ΔiMELD	0.825 (0.733–0.918)^d,i^	0.000	5.5	73.7	82.1	-	-	0.095
	0.805 (0.704–0.906)^e,h^	0.000	41.5	66.7	86.8	0.449	0.014	-
	0.861 (0.776–0.947)^e,i^	0.000	6.5	72.7	89.5	-	-	0.213
	0.762 (0.621–0.903)^f,h^	0.003	41.5	68.2	82.6	0.643	0.122	-
	0.767 (0.626–0.908)^f,i^	0.002	5.5	72.7	78.3	-	-	0.161
	0.871 (0.745–0.997)^g,h^	0.000	40.5	75.0	87.5	0.745	0.139	-
	0.900 (0.796–1.005)^g,i^	0.000	6.5	75.0	93.8	-	-	0.954
MELD-XI	0.647 (0.524–0.771)^d,h^	0.026	20.5	55.3	71.8	0.119	0.067	-
ΔMELD-XI	0.789 (0.680–0.898)^d,i^	0.000	4.5	65.8	89.7	-	-	0.044
	0.628 (0.496–0.759)^e,h^	0.065	-	-	-	0.028	0.077	-
	0.833 (0.728–0.938)^e,i^	0.000	4.5	69.7	92.1	-	-	0.131
	0.597 (0.430–0.763)^f,h^	0.266	-	-	-	0.189	0.129	
	0.757 (0.614–0.899)^f,i^	0.003	4.5	54.5	87.0	-	-	0.081
	0.725 (0.540–0.910)^g,h^	0.030	20.5	62.5	71.2	0.596	0.148	-
	0.805 (0.614–0.995)^g,i^	0.003	4.0	81.2	93.8	-	-	0.213
MELD-Na	0.769 (0.659–0.878)^d,h^	0.000	23.5	71.1	84.6	0.412	0.046	-
ΔMELD-Na	0.834 (0.745–0.924)^d,i^	0.000	4.5	73.7	84.6	-	-	0.202
	0.756 (0.639–0.874)^e,h^	0.000	23.5	66.7	86.8	0.235	0.043	-
	0.858 (0.770–0.947)^e,i^	0.000	4.5	75.8	86.8	-	-	0.135
	0.736 (0.586–0.887)^f,h^	0.007	23.5	68.2	82.6	0.696	0.189	-
	0.782 (0.645–0.918)^f,i^	0.001	4.5	72.7	78.3	-	-	0.433
	0.826 (0.672–0.980)^g,h^	0.002	23.0	75.0	87.5	0.530	0.193	-
	0.895 (0.784–1.005)^g,i^	0.000	5.0	75.0	93.8	-	-	0.753
UKMELD	0.766 (0.658–0.874)^d,h^	0.000	45.5	57.6	81.6	0.753	0.215	-
ΔUKMELD	0.792 (0.691–0.892)^d,i^	0.000	3.5	63.2	84.6	-	-	0.077
	0.763 (0.649–0.876)^e,h^	0.000	45.5	60.5	79.5	0.536	0.127	-
	0.818 (0.718–0.918)^e,i^	0.000	3.5	66.7	86.8	-	-	0.100
	0.735 (0.588–0.883)^f,h^	0.007	45.5	50.0	82.6	0.846	0.329	-
	0.711 (0.559–0.864)^f,i^	0.015	3.5	59.1	83.3	-	-	0.097
	0.820 (0.668–0.972)^g,h^	0.002	45.5	75.0	75.0	0.398	0.622	-
	0.904 (0.804–1.005)^g,i^	0.000	4.5	68.8	100	-	-	0.967

Comparisons were performed ^a^within each pair of models; ^b^to the traditional MELD score; ^c^to the ΔMELD score. AUCs were calculated for ^d^all patients with ACHBLF (n=77); ^e^patients with MELD score ≤30 (n=71); ^f^patients treated with antivirals (n=45); ^g^patients not treated with antivirals (n=32). ^h^AUCs for MELD series; ^i^for respective delta (Δ) scores. MELD, model for end-stage liver disease; AUC, area under the receiver operating characteristic (ROC) curve.
